# Scalable fabrication of a hybrid field-effect and acousto-electric device by direct growth of monolayer MoS_2_/LiNbO_3_

**DOI:** 10.1038/ncomms9593

**Published:** 2015-10-23

**Authors:** Edwin Preciado, Florian J.R. Schülein, Ariana E. Nguyen, David Barroso, Miguel Isarraraz, Gretel von Son, I-Hsi Lu, Wladislaw Michailow, Benjamin Möller, Velveth Klee, John Mann, Achim Wixforth, Ludwig Bartels, Hubert J. Krenner

**Affiliations:** 1 Chemistry, Materials Science & Engineering and Electrical Engineering, University of California, Riverside, California 92521, USA; 2 Lehrstuhl für Experimentalphysik 1 and Augsburg Centre for Innovative Technologies (ACIT), Universität Augsburg, Universitätsstrasse 1, Augsburg 86159, Germany; 3 Nanosystems Initiative Munich (NIM), Schellingstrasse 4, München 80799, Germany; 4 Department of Physics, Pepperdine University, 24255 Pacific Coast Highway, Malibu, California 90263, USA; 5 Center for NanoScience (CeNS), Ludwig-Maximilians-Universität München, Geschwister-Scholl-Platz 1, München 80539, Germany

## Abstract

Lithium niobate is the archetypical ferroelectric material and the substrate of choice for numerous applications including surface acoustic wave radio frequencies devices and integrated optics. It offers a unique combination of substantial piezoelectric and birefringent properties, yet its lack of optical activity and semiconducting transport hamper application in optoelectronics. Here we fabricate and characterize a hybrid MoS_2_/LiNbO_3_ acousto-electric device via a scalable route that uses millimetre-scale direct chemical vapour deposition of MoS_2_ followed by lithographic definition of a field-effect transistor structure on top. The prototypical device exhibits electrical characteristics competitive with MoS_2_ devices on silicon. Surface acoustic waves excited on the substrate can manipulate and probe the electrical transport in the monolayer device in a contact-free manner. We realize both a sound-driven battery and an acoustic photodetector. Our findings open directions to non-invasive investigation of electrical properties of monolayer films.

## Abstract (web summary)

Lithium niobate has piezoelectric and birefringent properties useful for optics, but it is not readily integrated with traditional optoelectronics. Here, the authors create a device that senses photoconductance in semiconducting molybdenum disulfide using surface acoustic waves excited in lithium niobate.

## Graphical abstract

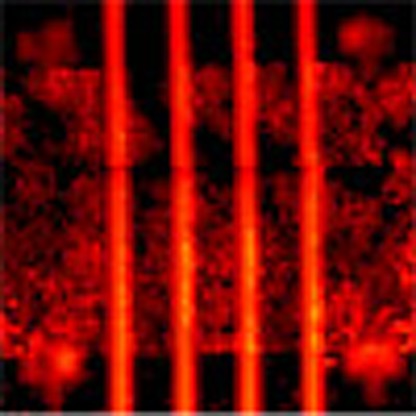

Through a combination of substantial piezoelectric and birefringent properties, LiNbO_3_ offers pronounced electro-optic, acousto-optic and acousto-electric activities^1^ that render it the material of choice for numerous applications in radio frequency (RF) signal processing^2^,^3^ and passive integrated optics^4^. LiNbO_3_, the ‘silicon of photonics'^5^,^6^, exhibits a large, ∼3.95 eV indirect bandgap^7^. For application as an optical sensor, hybridization with a lower bandgap material is of key relevance. Here we report millimetre-scale direct chemical vapour deposition of monolayer MoS_2_ onto 128°YX-cut LiNbO_3_. Field-effect transistors (FETs) fabricated on these films exhibit characteristics competitive with established transition metal dichalcogenide (TMD) devices on silicon^8^,^9^,^10^,^11^. The acousto-electric activity of the LiNbO_3_ substrate permits concomitant control and measurement of the systems electronic and optical properties in a contact-free manner. In our hybrid device, surface acoustic waves (SAWs) excited directly on the LiNbO_3_ substrate induce a strong acousto-electric effect and sense remotely the photoconductance of an TMD monolayer. SAW photoconductance spectroscopy can be performed at any point along the propagation path of the wave that extends on the millimetre length scale of a chip. This is in strong contrast to contact-based transport measurements, for which only the sample area between the contacts can be probed.

TMD films have attracted considerable attention for opto-electronic applications because of their direct-bandgap-semiconducting property at the single-layer limit^12^,^13^. Offering a tunable bandgap of 1.1–1.9 eV (MoTe_2_ to WS_2_), these two-dimensional (2D) semiconductors can complement the properties of graphene through their strong photoluminescence (PL), significant spin–orbit coupling^14^,^15^, and ensuing valleytronics physics^16^,^17^. Numerous reports have shown functional FET devices of MoS_2_ and other TMDs^8^,^9^,^10^,^11^; prototypical TMD FETs have been applied for gas sensing^18^, extended to ferroelectric gating^19^,^20^ and were used to construct a memory element^21^,^22^. So far, most investigations have focused on conventional semiconductor platforms, typically utilizing TMD material exfoliated onto SiO_2_/Si substrates. A range of chemical vapour deposition-based growth methods has been developed for TMD films on different substrates, including SiO_2_, sapphire, and graphene^23^,^24^,^25^,^26^,^27^,^28^,^29^,^30^. LiNbO_3_ is routinely used in numerous commercial SAW devices, ranging from RF filters to wirelessly interrogated and identity-tagged devices, as well as in microfluidics. In addition, LiNbO_3_ is the material of choice for integrated photonic devices that harness its native nonlinear optical properties for classical and quantum communication. Hence, the hybridization of TMDs with 128°YX-cut LiNbO_3_ is a key technological advancement.

Acousto-electric spectroscopy has a long-standing tradition in probing and controlling solid-state materials^31^,^32^. SAWs in particular offer a versatile approach since these nanoscale sound waves can be excited and detected all-electrically on a chip. SAW spectroscopy has been performed on bulk semiconductors and their heterostructures even in the quantum regime^33^,^34^,^35^. For more than 15 years, semiconductor-LiNbO_3_ hybrids have been realized by epitaxial lift-off and transfer onto the LiNbO_3_ substrate^36^. Recently, similar hybrids have been reported for graphene^37^,^38^,^39^,^40^. These applications have in common that the semiconducting layer is loosely attached to the substrate via weak van der Waals' interactions. This interaction preserves contact-free acousto-electric access but its ability to offer acousto-mechanical couplings has not been determined.

A particularly exciting aspect of transport measurements using SAWs is their inherently contact-free nature combined with their sensitivity in the limit of low conductivity. The transport properties reported for TMD materials, such as transconductance, carrier mobility and susceptibility to gating, vary widely even for measurements on the same TMD material. From the perspective of technological application, this is very undesirable. In particular, charge transfer at the interface between metal contacts and the 2D TMD films, resulting in band shifting/bending analogue to the formation of a Schottky barrier, has been reported to affect transport measurements^41^,^42^,^43^. SAW-based devices will ultimately have the power to provide entirely contact-free measurements, thus opening a new avenue to shed light on this current issue.

Here we report on two technological advancements: the scalable fabrication of a hybrid MoS_2_/LiNbO_3_ FET-electro-acoustic device that combines FET functionality with response to SAWs and the cross-validation of the respective signals; we demonstrate the versatility and power of this approach by measurement of the photoconductivity of a single-layer MoS_2_ film.

## Results

### Device layout and characteristics

**Figure 1:**
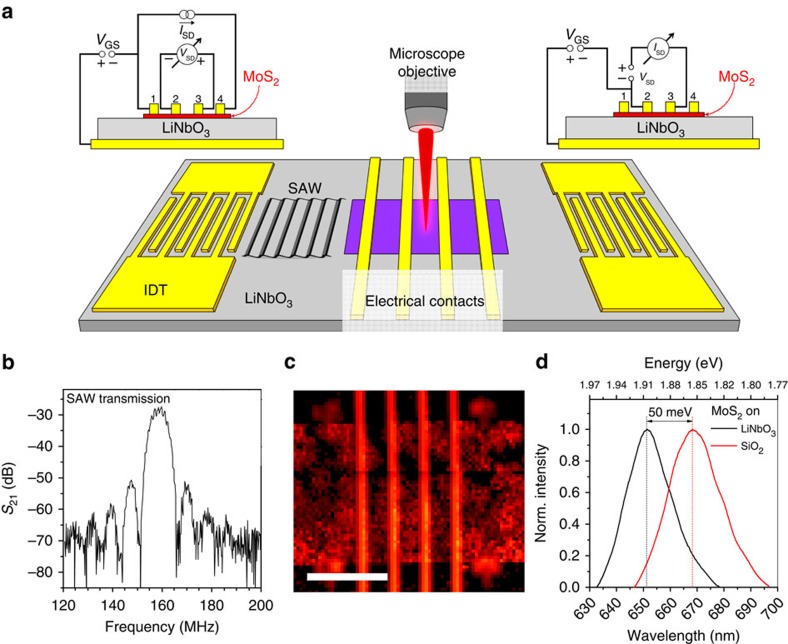
Sample. (**a**) Schematic representation of our hybrid MoS_2_/LiNbO_3_ device. Four Ti/Au electrodes form the contacts of a FET fabricated on chemical vapour deposition-grown MoS_2_. Two opposing, non-impedance-matched IDTs are used to excite SAWs propagating across the MoS_2_ FET. The insets show the electrical wiring configurations for 4-point (left) and 2-point (right) measurements. The sample was excited optically using a × 50 microscope objective with a numerical aperture (NA) of 0.55. (**b**) SAW transmission between the IDTs across the FET device shows a pronounced 40 dB maximum at the design frequency *f*_SAW_=160 MHz of the 5.4-mm long delay line. (**c**) PL map of the active FET region (scale bar, 100 μm). Monolayer MoS_2_ PL intensity (colour coded: red high intensity, black low intensity) is detected only in the channel region. Reflection from the FET contacts and alignment marks is clearly visible. (**d**) Comparison of single-point PL spectra obtained on SiO_2_ (red) and our 128°YX-cut LiNbO_3_ substrate (black) reveals a blueshift attributed to compression of the MoS_2_ film. Norm., normalized.

A schematic of our hybrid device is shown in Fig. 1a and the fabrication procedure is summarized in the Methods section. The device consists of two components: (i) a SAW delay line formed by a pair of interdigital transducers (IDTs) and (ii) a monolayer MoS_2_-FET centred in between the two IDTs. This configuration enables us to probe and manipulate the electrical characteristics of the FET by exciting and detecting SAWs interacting with carriers in the MoS_2_. IDTs are used for all-electrical excitation and detection of SAWs. On LiNbO_3_, SAWs propagate at a velocity *v*_SAW_=3,980 m s^−1^; our IDTs are designed for a frequency of *f*_SAW_=160 MHz corresponding to a design wavelength *λ*_SAW_=25 μm. Their arrangement allows measurement of the scattering parameter, *S*_21_, that is, the SAW transmission from one IDT to the other. In Fig. 1b we plot *S*_21_ as a function of the RF signal applied to the sending IDT. In this trace, the delay line resonance frequency is resolved as a 40-dB high transmission maximum very close to the nominal design frequency of *f*_SAW_*=*160 MHz. This RF characterization demonstrates high efficiency generation, transmission and detection of SAWs on the LiNbO_3_ host substrate even after its exposure to the MoS_2_ growth conditions. FET fabrication was performed on a monolayer region of the as-grown MoS_2_ film; its single-layer thickness was validated by scanning PL spectroscopy. An overview map demonstrating millimetre-scale growth of monolayer MoS_2_ onto 128°YX-cut LiNbO_3_ is presented in Supplementary Fig. 1. After PL characterization, a 4-terminal MoS_2_ FET is monolithically defined on the LiNbO_3_ substrate in the region of maximum emission of monolayer MoS_2_. The FET is fabricated to be located in between a pair of IDTs. Figure 1c shows a 400 μm × 350 μm spatial map of the characteristic monolayer MoS_2_ PL emission in the channel region^12^,^13^ of the FET. The PL intensity is encoded in colour scale with red/dark regions corresponding to high/low count rates, respectively. PL mapping is used to determine the extent of the monolayer film before device fabrication and it confirms the presence of a monolayer MoS_2_ channel in the completed FET. Intensity variations are likely due to variations in the film domain size as shown in ref. 25. In all other areas, the MoS_2_ film was selectively removed to avoid any signal contributions from these regions. The four vertical lines indicate reflection from the Au contact lines; the four diffuse spots close to the corners of the panel represent gold alignment marks used during fabrication of the FET channel. The upper and lower boundaries of the FET channel in Fig. 1c are aligned with the SAW propagation path. Thus, we ensure tight correspondence of the electrically and acoustically addressed film area. The LiNbO_3_ substrates serves as the 
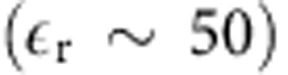
 dielectric for back gating to ensure full FET operation and we provide full electrical characterization of this device. We show the respective wiring diagrams for 4- and 2-point configuration as insets in Fig. 1a.

In Fig. 1d, we compare normalized PL spectra from MoS_2_ films grown under nominally identical conditions on LiNbO_3_ (black) and on a SiO_2_/Si reference substrate (red). For both samples, a PL peak is clearly resolved, corroborating the monolayer nature of the MoS_2_ (Supplementary Fig. 2 also provides Raman spectroscopy). However, MoS_2_ grown on 128°YX-cut LiNbO_3_ yields PL signal at photon energies larger by 40–50 meV; we attribute this shift to the fivefold larger thermal expansion coefficient of LiNbO_3_ (ref. 44) of 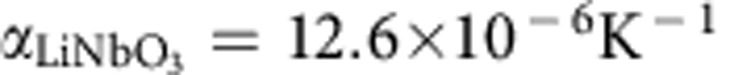
compared with that of Si (ref. 45) of 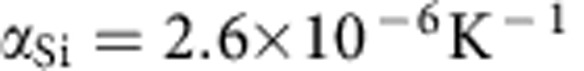 near room temperature. These dissimilar values cause a net relative compressive strain of 0.35% for the MoS_2_ film on LiNbO_3_ during cool-down from growth temperatures. Compressive strain is expected to give rise to a blueshift of the PL emission. Extrapolating the data on uniaxial tensile stress reported in ref. 46 of Δ*E*=60–70 meV per 1% strain, we expect a blueshift of ∼23 meV, about ½ of the observed blueshift. Using the value of Δ*E*=300 meV per 1% strain reported by Hui *et al*.^47^ for compressive strain in trilayer MoS_2_, the expected blueshift amounts to 105 meV, which is larger than the value observed here. Thus, our observation of a blueshift is compatible in magnitude with recent work and corroborates a rigid connection of the MoS_2_ film to the LiNbO_3_ substrate, a precondition for maximum interaction with the SAW.

### FET characterization

**Figure 2:**
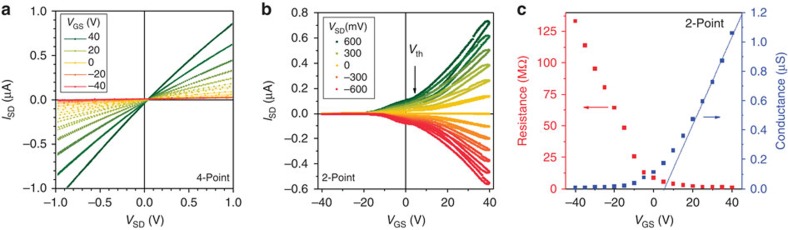
FET operation of hybrid MoS_2_/LiNbO_3_ device. (**a**) Output characteristics (*I*_SD_ versus *V*_SD_) for different gate voltages *V*_GS_ recorded in 4-point configuration. For large negative *V*_GS_, the device is weakly conducting; an *n*-type channel is formed for positive *V*_GS_. (**b**) Transfer characteristics (*I*_SD_ versus *V*_GS_) for different source-drain voltages *V*_SD_ recorded in 2-point configuration shows pronounced increase of |*I*_SD_| at positive *V*_GS_ due to formation of an *n*-type channel. (**c**) Channel resistance (red) and conductance (blue) as a function of *V*_GS_ extracted from 2-point output characteristics at *V*_SD_=0. For positive *V*_GS_, a linear fit indicates a mobility *μ*_FE_=33±5 cm^2^ V s^−1^ and a threshold voltage *V*_th_=5.5±1.5 V. The latter agrees well with that derived from the data in **b**, as summarized in Table 1.

We expect our hybrid device to exhibit FET properties. Owing to the very large dielectric constant *ɛ*_r_ ∼50 for this cut of LiNbO_3_, moderate electric fields *D*=*ɛ*_r_*V*_GS_/*d*_sub_=±40 kV cm^−1^ can be achieved by applying *V*_GS_=±40 V between the LiNbO_3_ backside (thickness *d*_sub_=500 μm) and the MoS_2_ layer. We test FET operation of our hybrid device by measurement of its transport characteristics as a function of a back gate voltage (*V*_GS_) across the LiNbO_3_ substrate. In Fig. 2a, we plot a set of output characteristics (*I*_SD_ versus *V*_SD_), recorded in 4-point configuration, for different back gate voltages ranging between *V*_GS_=±40 V. As *V*_GS_ is tuned from negative to positive polarity, we observe the expected reduction of the sheet resistance due to accumulation of electrons in the MoS_2_ monolayer. This observation clearly demonstrates FET operation in our hybrid device in the linear regime and the formation of an *n*-type transport channel, which is in agreement with prior work^8^,^9^,^10^,^11^. Transfer characteristics (*I*_SD_ versus *V*_GS_) recorded in 2-point configuration are plotted in Fig. 2b for different *V*_SD_. Again, the increase of *I*_SD_ as *V*_GS_ is tuned to positive bias at constant *V*_SD_ is consistent with *n*-type character. We note that in the data, a small leakage current through the substrate was subtracted for clarity; Supplementary Fig. 3a contains the graphs before subtraction of gate leakage. The transfer characteristics exhibit a small hysteresis, which may arise from poling effects of LiNbO_3_ at the interface to the MoS_2_ layer^19^. We note that MoS_2_-based FETs on dielectric Si/SiO_2_ substrates have been found to be sensitive to the local environment and, in particular, to the surrounding gas atmosphere^18^. Adsorption and desorption of impinging gas atoms and molecules have been suggested as an origin of an hysteretic *I–V* characteristics^10^. From the turn-on behaviour, we are able to derive the threshold voltage *V*_th_, marked in Fig. 2b. From this analysis, we can determine the field-effect mobility in our device given by 
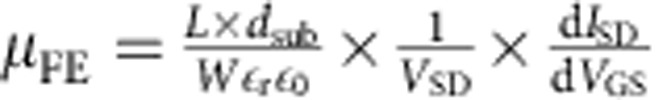
, with *L* and *W* being the length and width of the channel, respectively and *d*_sub_ denotes the thickness of the LiNbO_3_ substrate. An example analysis is presented in Supplementary Fig. 3b, and the *V*_SD_ dependence of the values *V*_th_ and *μ*_FE_ are presented in Supplementary Fig. 3c and d, respectively. From the output characteristics set, we are able to extract the channel's electrical properties as a function of *V*_GS_. Figure 2c depicts the channel resistance and conductance extracted from 2-point output characteristics evaluated at *V*_DS_=0 and plotted versus *V*_GS_. Moreover, from a linear fit of the conductance for *V*_GS_>20 V, we can determine *μ*_FE_ and *V*_th_ from the slope and from the intersection at *I*_SD_=0, respectively. We performed an analogous analysis on the 4-point output characteristics that is presented in Supplementary Fig. 4. Table 1 summarizes the results for *μ*_FE_ and *V*_th_; the error estimates originate from the accuracy of the linear fit and from the s.d. of 12 *V*_GS_ up- and down-sweeps for output and transfer characteristics, respectively. The values for *μ*_FE_ and *V*_th_ derived from these independent sets of data are in good agreement. We note that the values obtained on our highly piezoelectric architecture are competitive with back-gated devices fabricated by exfoliation on the SiO_2_/Si platform^48^.

### Acousto-electric effect

**Figure 3:**
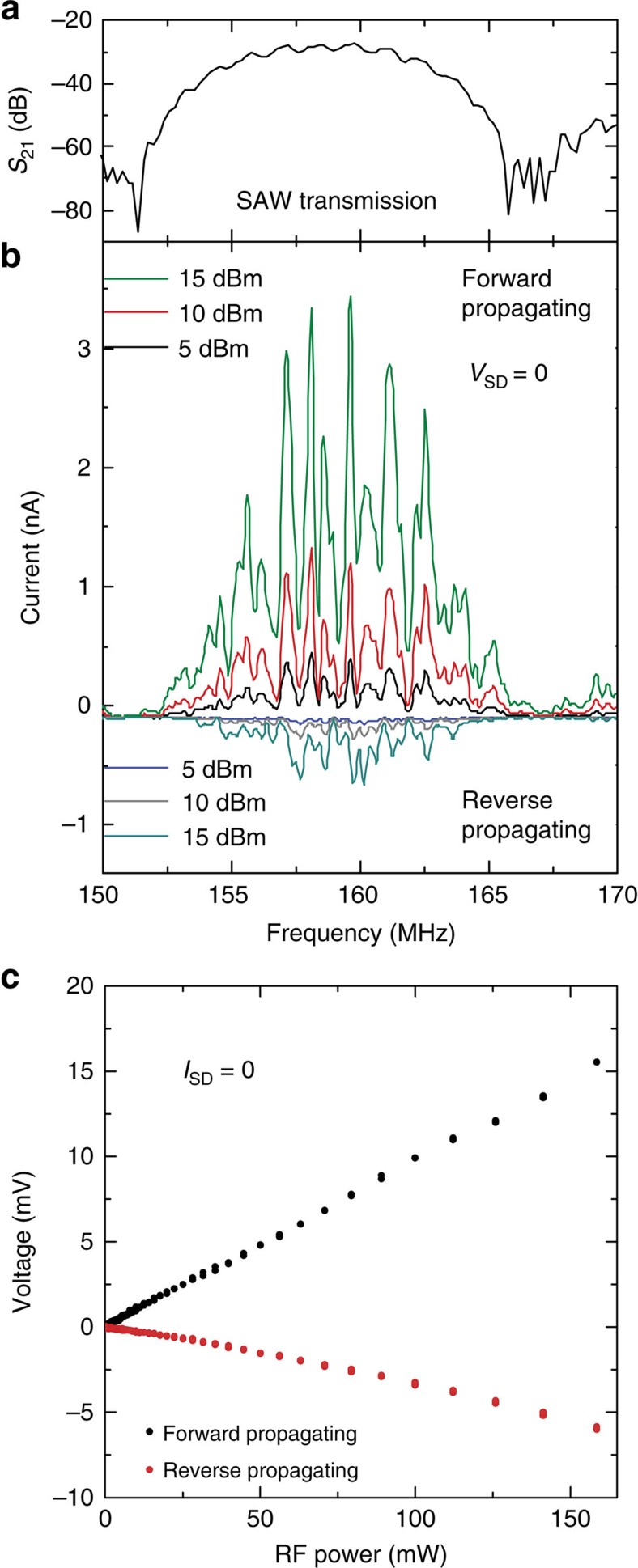
Acousto-electric spectroscopy. (**a**) Frequency band of the SAW transmission between IDTs plotted as the scattering parameter *S*_21_. (**b**) AEC as a function of RF applied to the IDTs for different RF power levels *P*_RF_. Current measurements were performed in a 2-point short-circuit (*V*_SD_=0) configuration. The forward and reverse propagating SAWs were excited by either of the two opposing IDTs. They yield AECs of opposite sign. (**c**) AEV as a function of *P*_RF_ measured in 4-point, open-circuit configuration (*I*_SD_=0). For both SAW propagation directions, the expected linear dependence is well reproduced. The signs of the AECs and voltages correspond to *n*-type conductivity of the film.

Having established the electrical functionality of our hybrid device, we validate its acousto-electric transport properties. Acousto-electric effects are expected within the transmission band of the SAW delay line. The frequency dependence of the corresponding scattering parameter *S*_21_ is plotted in Fig. 3a. First, we measure the short-circuit (*V*_SD_=0) acousto-electric current (AEC; Fig. 3b) in a two-probe configuration as a function of an RF signal of varying frequency and power *P*_RF_ applied to each of the two IDTs. For a forward propagating SAW excited by constant *P*_RF_, we observe that the AEC exhibits a characteristic frequency dependence. This dependence faithfully reproduces the *S*_21_ data. As the propagation direction of the SAW is reversed, the polarity of the AEC reverses: this finding indicates that the propagation direction of the SAW determines the direction of the carrier flow between the two contacts (that is, momentum transfer between the SAW and the mobile carriers in the MoS_2_ film). The observed polarities provide an independent verification of *n*-type majority charge carriers in the MoS_2_ film. We note that the different AEC levels measured for the two IDTs at constant *P*_RF_ arise from a combination of variations in their absolute conversion efficiencies, different distances from the location of our measurements and different SAW attenuation along the propagation path. The lower amplitudes of the AEC compared with reports on graphene on LiNbO_3_ (refs 37, 38) are expected due to the lower carrier concentrations in our MoS_2_ films compared with zero-bandgap graphene.

Second, we explore the *P*_RF_ power dependence of the acousto-electric effect for both SAW directions in a complementary experiment by measuring the open-circuit voltage in a 4-point configuration (Fig. 1b) with open connection to the back contact. Here the total current between the two outer contacts is set to *I*_SD_=0 and the acousto-electric voltage (AEV) is picked up between the two inner contacts. In Fig. 3c, we plot the measured AEV as a function of *P*_RF_ (in mW) applied for a forward (black) and reverse (red) propagating SAW. We observe the expected linear power dependence of the acousto-electric effect. We note that the AEC and voltage represent sound-driven constant current and voltage sources, respectively. Such an ‘acoustic battery' is remotely driven by the SAW.

### Contact-free photoconductivity probe

Having established the functionality of our hybrid device, we proceed by applying it to the measurement of the photoconductivity of the MoS_2_ layer. MoS_2_ single-layer material exhibits a pronounced photoconductive response to optical (above bandgap) irradiation^49^,^50^,^51^. SAWs provide an extremely sensitive and fast conductivity (*σ*) probe and are particular suitable to the characterization of poorly conductive films^52^. The SAW attenuation coefficient is given by:


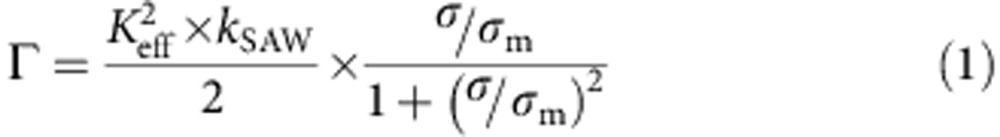


In this expression, 
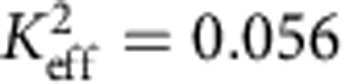
 is the electromechanical coupling efficiency, *k*_SAW_ is the SAW wave vector and 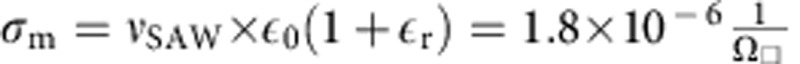 is the characteristic sheet conductivity. This value corresponds to a characteristic channel conductance of 
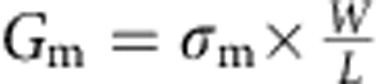
 ∼18.5 μS in our device. This value is larger than the measured channel conductance *G*<1.2 μS derived from FET the characteristics of Fig. 2.

**Figure 4:**
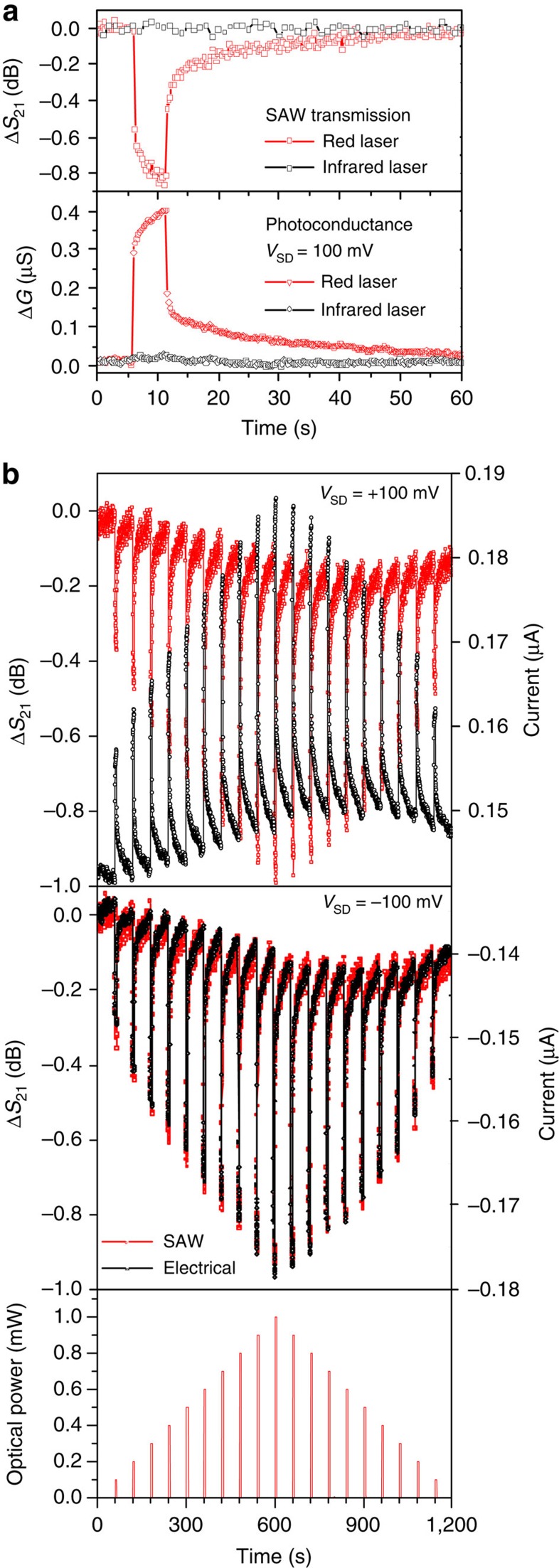
Photoconductance spectroscopy. (**a**) Comparison of the time-dependent photoresponse detected by the change of the transmitted SAW intensity (Δ*S*_21_) with the change of the 2-point conductance (Δ*G*) of the FET. Red and black traces were recorded for *P*_laser_=1 mW excitation by a red and infrared laser, respectively. These lasers are switched on for Δ*t*=5 s at *t*=6 s. Both the instantaneous and persistent features of the photoresponse are consistently resolved by both measurement techniques. For excitation with an infrared laser, no photoresponse is detected, proving that the signal detected for the red laser indeed stems from the MoS_2_ monolayer. (**b**) Comparison of Δ*S*_21_ with *I*_SD_ under photoexcitation using a red laser. The laser is switched on every 1 min for Δ*t*=5 s. Each successive minute *P*_laser_ is increased by 0.1 mW until *P*_laser_=1 mW is reached. Subsequently, *P*_laser_ is decreased to 0 mW in steps of Δ*P*_laser_=100 μW as shown in the lower panel. Upper and centre panel compare the SAW transmission (Δ*S*_21_, red) and photocurrent (*I*_SD_, black) for *V*_SD_=+100 mV and −100 mV, respectively. Direct correspondence between Δ*S*_21_ and *I*_SD_ is confirmed: while Δ*S*_21_ reduces irrespective of voltages, the sign of *I*_SD_ is determined by the polarity of *V*_SD_.

To characterize the SAW transmission along the delay line, we measured the *S*_21_ scattering parameter, that is, the transmitted SAW intensity from one IDT to the other. In the upper panel of Fig. 4a, we plot the variation of *S*_21_ as a function of time. At *t*=6 s, a diffraction-limited spot in the centre of the hybrid device is irradiated for Δ*t*=5 s by either a red (*hν*=1.87 eV) or an infrared laser (*hν*=1.46 eV) source at 1 mW power. The red laser is resonant with the fundamental optical transition of MoS_2_ on LiNbO_3_, while the photon energy of the infrared laser is less than the optical bandgap of MoS_2_. We resolve a pronounced photoresponse for the red laser, manifesting itself in a reduction of the transmitted SAW signal (Δ*S*_21_ <0). Such an increase of the attenuation is expected from equation (1) since photogeneration of electrons and holes leads to an increase of *G*, while still remaining in the *G*<<*G*_m_ regime. For the infrared laser, no variation of the SAW attenuation was resolved, corroborating that the observed response indeed arises from photogenerated carriers in the MoS_2_. We confirm this interpretation by simultaneously measuring the electrical conductivity in 2-point configuration, which is plotted in the lower panel of Fig. 4a. The anticipated increase of the conductance (Δ*G*>0) is clearly resolved. The agreement of the global features of Δ*S*_21_ and Δ*G* is remarkable: both channels show quasi-instantaneous responses as the laser is switched on and off, which is attributed to the presence or absence of photogenerated carriers in the MoS_2_ layer. The associated processes occur on a timescale faster than the acquisition time of each data point of 250 ms. In addition to this fast contribution, a response on longer (seconds to minutes)^50^ timescales is resolved clearly.

In Fig. 4b, we present a detailed optical pump power series. In this experiment, the laser source is repeatedly switched on for 5 s every minute. The optical pump power is initially increased in Δ*P*_laser_=0.1 mW steps from *P*_laser_=0.1–1 mW and then reduced to *P*_laser_=0. The corresponding optical power pattern is plotted in the lower panel of Fig. 4b. The upper and centre panels compare the measured SAW (Δ*S*_21_) and current (*I*_SD_) responses for *V*_SD_=+100 mV and −100 mV, respectively. Clearly, both the SAW attenuation and the FET current scale with the laser power in a nonlinear manner similar to the observations of Yin *et al*.^49^ and Lopez-Sanchez *et al*.^51^ but different from the short-channel devices of ref. 50. While the sign of *I*_SD_ depends on the polarity of *V*_SD_, Δ*S*_21_ decreases irrespective of the *V*_SD_ polarity. Furthermore, the amplitude of Δ*S*_21_ only depends on *P*_laser_ and is independent of the applied *V*_SD_. These facts prove that electro-acoustic and FET operation do not interfere. The component of the photoresponse with the longer time constant^50^ leads to the accumulation of higher sheet conductivity over the full 20-min duration of the experiment. Such processes are frequently observed in 2D materials and are typically attributed to traps at the interface to the substrate or in the material itself. In ref. 50, we demonstrate the composition dependence of this phenomenon for MoS_2(1-*x*)_Se_2*x*_ alloys.

## Discussion

Direct growth of MoS_2_ onto the 128°YX-cut of LiNbO_3_ permits acousto-electric spectroscopy on the TMD overlayer as validated by the hybrid device assembled in this work. This finding opens many new avenues of research: while our hybrid device relied on metal contacts to the TMD film so as to validate congruence between electric transport and SAW-based conductivity measurements, subsequent experiments may dispense with the contacts, thereby allowing entirely contact-free transport measurements on TMD films. Moreover, the tight coupling of the TMD film to the underlying substrate as being indicated by the blueshift of the PL signal suggests that not only acousto-electric but also acousto-mechanic spectroscopy on TMD films may be possible. In such experiments, the SAW exerts tensile or compressive strain allowing measurement of the coupling of the dynamic deformation to the electronic degrees of freedom of the TMD material. Spin and charge excitations in recently discovered TMD-based quantum dots^53^ could also be controlled dynamically by SAW-driven deformation potential coupling and Stark effect^54^. The rigid connection of the TMD layer to the LiNbO_3_ is crucial for such acousto-mechanically driven approaches, since it ensures close coupling of the SAW to the film. We also highlight that our device fabrication used exclusively scalable techniques avoiding transfer or exfoliation steps. This paves the road towards the incorporation of TMD films as, for example, optically active elements, into conventional and inexpensive LiNbO_3_-based SAW devices of a type similar to those currently used, for example, as frequency filters in cell phones. As a consequence, we foresee that the fundamental device concept introduced in this article will attain widespread application both in the fundamental study of the properties of TMD films and in the technological realm where optically active thin, inorganic and durable films are desired: our SAW device remained functional for 9 months in air withstanding multiple intermittent thermal cycles of heating to temperatures as high as >450 K and cooling to as low as <10 K in vacuum in the meantime. Measurements on different TMD materials show promising initial results and will be reported on once completed.

## Methods

### Sample fabrication

*TMD growth*. Single-layer MoS_2_ synthesis follows the technique outlined in ref. 25: in a typical setup, 25 mg of molybdenum trioxide powder (99.99%, Sigma-Aldrich) was placed in an alumina boat and centred in the tube furnace. The LiNbO_3_ substrate (128°YX-cut, oxygen reduced and weakly conductive ‘black' LiNbO_3_, thickness *d*_sub_=500 μm) was mounted with a molybdenum mesh (Alfa Aesar) on the edge of the boat and 1 g of sulfur was placed upstream at a distance of 25 cm from the centre. The furnace was heated at a rate of 12.5 °C min^−1^, held at ∼650 °C for 20 min and then allowed to cool naturally to room temperature. N_2_ carrier gas aided the transfer of the sulfur vapour to the sample region for optimal growth.

*Device layout and fabrication*. After PL identification of the region with monolayer growth, we fabricated Ti/Au metal contacts and IDTs (split-2 design, 21 finger pairs, duty cycle 1:1, aperture 200 μm) for electric and acoustic interfacing to the MoS_2_ film, respectively. The IDT's design wavelength was chosen as *λ*_SAW_=25 μm, corresponding to a design frequency of *f*_SAW_=160 MHz. Supplementary Fig. 5 shows a micrograph of an IDT. As shown in Fig. 1a, the two IDTs are located at opposite ends of the substrate in a delay line configuration (length 5.4 mm) to enable us to launch and record SAWs propagating at *v*_SAW_=3,980 m s^−1^ in opposite directions across the chip. The studied FET device features a channel length of *L*=35 μm and a total width of the conducting channel of *W*=360 μm. After fabrication of the electrical contacts, in a second lithographic step, an oxygen plasma treatment is used to remove the MoS_2_ single-layer film from the channel area except for a 180-μm-wide region across, which the SAW propagates. Thus, we ensure correspondence of the electrically and acoustically addressed film area. Figure 1c shows a PL map of the contact region after electrode deposition and removal of extraneous MoS_2_ film.

*Lithographic patterning*. All lithographic patterning of the IDTs and contact electrodes proceeded in a single exposure step using an electron beam writer and polymethyl methacrylate (PMMA) as resist. Contacts were fabricated by sequential deposition of 10 nm of Ti followed by 60 nm of Au in an e-beam evaporation tool. Subsequent lift-off defined the active structures. MoS_2_ was selectively removed in a second e-beam exposure step and subsequent oxygen plasma treatment at a plasma power of 200 W at a pressure of 500 mTorr for 13 s.

### Measurement techniques

*SAW excitation and SAW transmission experiments*. For measurement of the AEC and AEV, the output of an RF signal generator was amplified and connected to one of the IDTs. The RF characteristics of the IDTs and SAW transmission lines were characterized using a vector network analyser measuring scattering parameters of the RF network, in particular the scattering parameters *S*_11_ (reflection) and *S*_21_ (transmission and insertion loss).

*Electrical characterization*. 2-Point characterization was performed using a Keithley K2400 source meter unit (SMU). For 4-point characterization, a K2400 SMU was used only as a constant current source (no measurement probes connected) and the voltage at the potential probes was recorded directly by a K2000 digital multimeter. The gate voltage was applied by a Keithley K2600 SMU, which measured the gate leakage current at the same time. Short-circuit AEC and open-circuit AEV were recorded using a K2400 SMU with *V*_SD_=0 and *I*_SD_=0, respectively.

*Optical spectroscopy*. The photoconductivity experiments relied on red (660 nm) and infrared (850 nm) pulsed semiconductor lasers with a 80-MHz repetition rate and a pulse duration ≤100 ps. PL and Raman spectroscopy, as well as mapping utilized a Horiba LabRAM HR spectroscopy system using a 532-nm excitation laser and a 1,800 lines per mm grating.

## Additional information

**How to cite this article:** Preciado, E. *et al*. Scalable fabrication of a hybrid field-effect and acousto-electric device by direct growth of monolayer MoS_2_/LiNbO_3_. *Nat. Commun.* 6:8593 doi: 10.1038/ncomms9593 (2015).

## Supplementary Material

Supplementary Information Supplementary Figures 1-5

## Figures and Tables

**Table 1:** Field-effect mobility and threshold voltage of our FET device determined by different techniques.

	**2-Point output characteristics (*****V***_**SD**_**=0)**	**4-Point output characteristics (*****V***_**SD**_**=0)**	**2-Point transfer characteristics**
*μ*_FE_ (cm^2 ^V^−1^ s^−1^)	33±5	43±5	37±5
*V*_th_ (V)	5.5±1.5	5.2±1.5	5.8±2.5

FET, Field-effect transistor.
