# Supplementary material for: Scalable fabrication of a hybrid field-effect and acousto-electric device by direct growth of monolayer MoS2/LiNbO3
Source: Nat Commun. 2015 Oct 23;6:8593. doi: 10.1038/ncomms9593 (PMC4639816; doi:10.1038/ncomms9593)
Supplement: Supplementary Information — Supplementary Figures 1-5 [file ncomms9593-s1.pdf]

Supplementary Figures

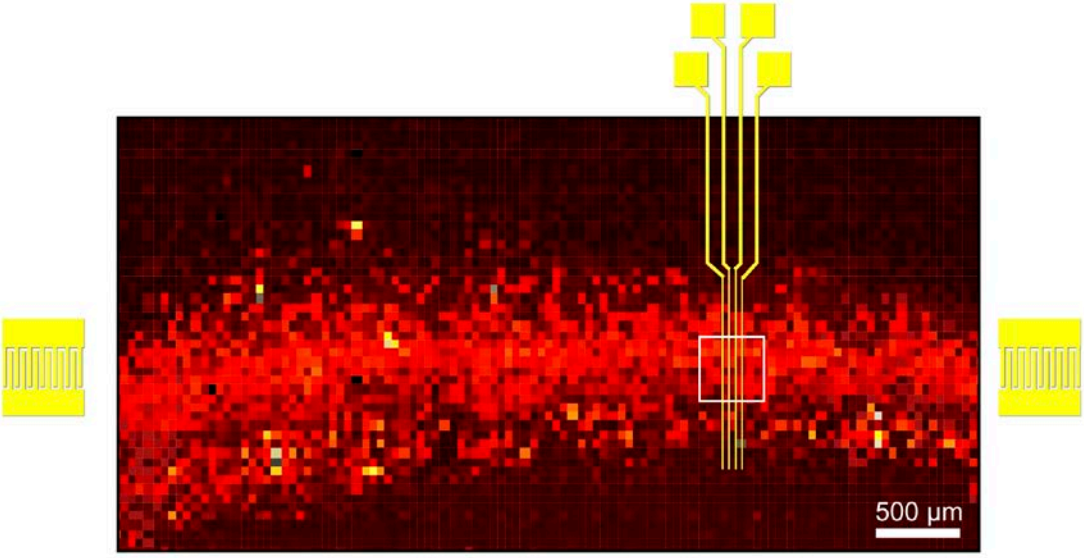

Supplementary Figure 1: Large area PL scan of monolayer MoS_2 signal. Intensity is color coded with red (dark) corresponding to high (low) count rates. These data demonstrate millimeter-scale CVD growth of monolayer MoS_2. In addition the positions of the used IDTs and the electrical contacts of the FET are shown.

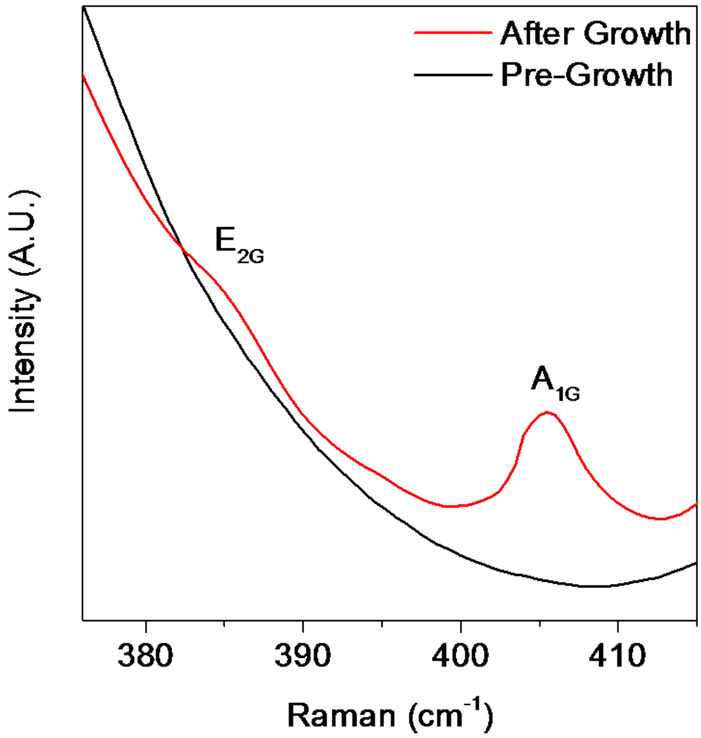

Supplementary Figure 2: Raman spectra of $LiNbO_3$ substrate before (black) and after (red) CVD growth of MoS_2. The additional signal of E_{2G} and A_{1G} modes confirms presence of MoS_2. Detailed analysis is hampered due to the dominant signal of the $LiNbO_3$ substrate.

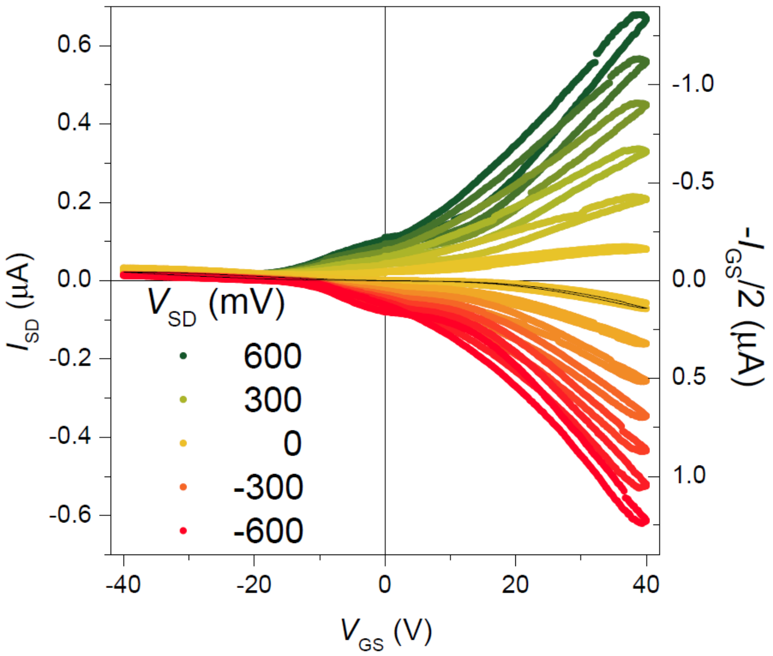

Supplementary Figure 3: Uncorrected FET output characteristics for different VSD (colored) and gate leakage current (black). The gate leakage current is weighted by a factor of 2.4 and subtracted from all characteristics. This procedure yields vanishing I_{SD} (V_{GS}) ~ 0 for V_{SD} = 0. The such obtained characteristics are shown in Fig. 2 (b) of the main paper.

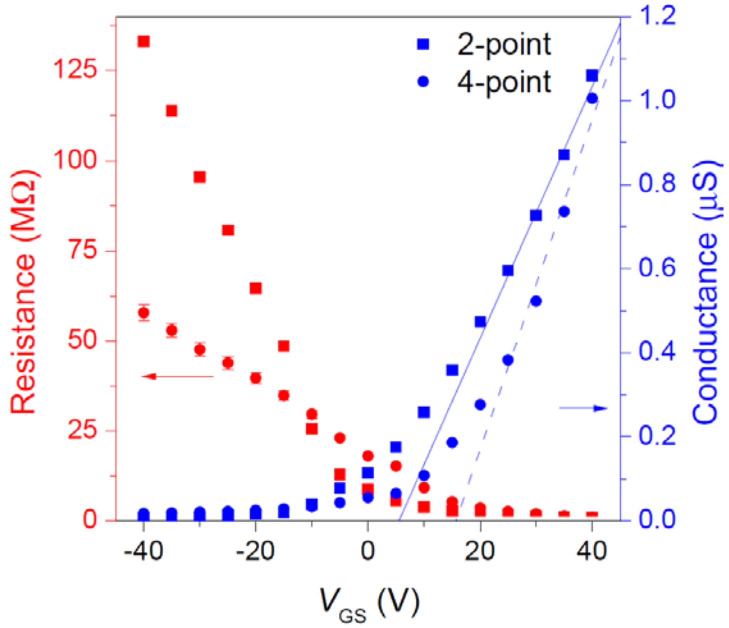

Supplementary Figure 4: Comparison of channel resistance (red) and conductance (blue) derived from 2-point (squares) and 4-point (bullets) data. The mobilities μ_{FE} for 2-point and 4-point measurements of 33 cm^2 V^{-1} s^{-1} and 43 cm^2 V^{-1} s^{-1}, respectively, are in good agreement. The threshold voltages $U_{th,2\text{-}point}$ = 5.5V derived from the 2-point measurements exceeds the $U_{th,4\text{-}point}$ = 15.7V derived from 4-point measurements by approximately a factor of 3 because of the 3fold increased total channel length in the latter configuration.

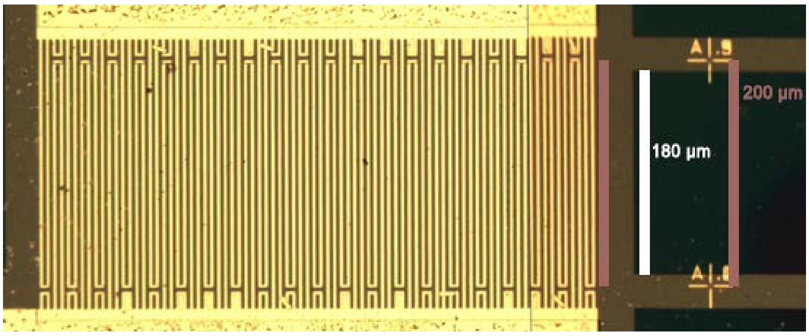

Supplementary Figure 5. Layout of the IDTs. The micrograph shows the double finger (Split-2) IDTs used in this study. They have 21 finger pairs, an aperture of 200 µm, a delay line width of 180 µm and they were fabricated 5.4 mm apart.
